# Adolescent idiopathic scoliosis: Indications and efficacy of nonoperative treatment

**DOI:** 10.4103/0019-5413.73655

**Published:** 2011

**Authors:** Federico Canavese, André Kaelin

**Affiliations:** University of Geneva Hospitals and Faculty of Medicine, Pediatric Orthopaedic Service, Department of Child and Adolescent, Rue Willy-Donzé, 6, 1211 Geneva 14, Switzerland

**Keywords:** Brace, conservative treatment, scoliosis, adolescents

## Abstract

The strategy for the treatment of idiopathic scoliosis depends essentially upon the magnitude and pattern of the deformity, and its potential for progression. Treatment options include observation, bracing and/or surgery. During the past decade, several studies have demonstrated that the natural history of adolescent idiopathic scoliosis can be positively affected by nonoperative treatment, especially bracing. Other forms of conservative treatment, such as chiropractic or osteopathic manipulation, acupuncture, exercise or other manual treatments, or diet and nutrition, have not yet been proven to be effective in controlling spinal deformity progression, and those with a natural history that is favorable at the completion of growth. Observation is appropriate treatment for small curves, curves that are at low risk of progression, and those with a natural history that is favorable at the completion of growth. Indications for brace treatment are a growing child presenting with a curve of 25°–40° or a curve less than 25° with documented progression. Curves of 20°–25° in patients with pronounced skeletal immaturity should also be treated. The purpose of this review is to provide information about conservative treatment of adolescent idiopathic scoliosis. Indications for conservative treatment, hours daily wear and complications of brace treatment as well as brace types are discussed.

## INTRODUCTION

The strategy for the treatment of idiopathic scoliosis depends essentially upon the size and pattern of the deformity, and its potential for progression. During the past decade, several studies have confirmed that the natural history of adolescent idiopathic scoliosis can be positively affected by nonoperative treatment, particularly bracing.^1^–^6^ The primary objective of nonoperative treatment is to successfully arrest progression of the curves or correct curves that cause or are likely to cause disability. Orthotic device selection is based on the type and level of curve and the anticipated tolerance of the patient. Avoidance of unnecessary surgery, cosmetic improvement and an increase of vital capacity, as well as pain control, are also of major importance.^7^–^14^

In 1985, the Scoliosis Research Society (SRS) initiated a controlled clinical trial to investigate the effectiveness of bracing as treatment for scoliosis. Patients of the same age, same curve pattern and severity were divided into two groups: one treated with bracing; and the other, untreated. Results published in 1993 demonstrated that brace treatment is effective compared to natural history.^2^ In another study,^3^ the records and radiographs of more than 1000 scoliotic patients treated by bracing were reviewed and compared with those of unbraced patients.^15^ This retrospective study confirmed that bracing is a more effective treatment to slow or arrest the progression of most spinal curvatures in skeletally immature patients in comparison with treatment not involving bracing. Furthermore, a meta-analysis of 20 studies showed that bracing 23 hours per day was significantly more successful than any other nonoperative treatment.^4^,^6^ Nevertheless, there are some patients for whom brace treatment is not effective.^16^

Other forms of nonsurgical treatment such as chiropractic or osteopathic manipulation, acupuncture, exercise or other manual treatments, or changes in diet and nutrition pattern have not yet been proven to be effective in controlling spinal deformities.

The purpose of this review is to summarize the available literature related to the conservative treatment of adolescent idiopathic scoliosis in relation to which patient to brace, when to stop bracing and type of brace.

## WHEN TO BEGIN TREATMENT

Observation is an appropriate treatment for small curves, curves that are at low risk of progression, and those with a natural history that is favorable at the completion of growth. Indications for brace treatment include, a growing child presenting with a curve of 25° to 40° or with curves less than 25° that have shown documented progression of 5° to 10° in six months (progression of more than 1° per month). Curves of 20° to 25° in those with pronounced skeletal immaturity (Risser, 0; Tanner, 1 or 2) should also be treated immediately. Braces should generally be worn full-time, with treatment lasting from two to four years, until the end of bone growth.^17^,^18^

By contrast, contraindication for bracing is, a child who has completed growth; or a growing child with a curve of over 45°, or less than 25° without documented progression.^2^,^3^,^6^,^19^ True thoracic lordosis is also a contraindication for orthotic treatment due to the effect of orthoses on the thoracic spine. A child with a nonsupportive home situation or who refuses to wear a brace should not be considered for brace treatment. Overweight adolescent patients will have greater curve progression and less success with bracing. In addition, the ability of a brace to transmit corrective forces to the spine through the ribs and soft tissue may be compromised in these patients.^20^

A prospective, multi-center study conducted by Nachemson *et al*. in several countries showed that the success rate of bracing was significantly higher compared to observation and surface electrical stimulation.^2^ A meta-analysis of 20 studies further supported this finding and showed that the weighted mean proportion of success was low for lateral electrical surface stimulation and for observation, and progressively higher for bracing at 8, 16 or 23 hours per day. The study concluded that bracing 23 hours per day was significantly more successful than any other treatment.^4^ Furthermore, a recently published systematic review concluded that bracing adolescent idiopathic scoliosis is effective in the long-term.^21^ However, it remains controversial as to whether or not a bracing program can decrease the frequency of surgery.^22^,^23^ A recently published systematic review used the number of surgically treated patients as an indicator of failure of bracing and reported a broad spectrum ranging from 1% to 43%.^24^,^25^

## DURING TREATMENT

When patients are first fitted with a brace, there is an initial adjustment period of usually one to two weeks. Initially, the patient is prescribed to wear the brace for a specific number of hours per day, and the orthosis is left slightly loose to allow the patient to gradually adjust to it. The brace is increasingly tightened daily until the appropriate level of snugness is reached. If any areas of tenderness or skin irritation develop, the brace is adjusted for optimal fit. Roentgenograms are performed after four weeks with the brace in place to verify the fit and determine the degree of curve reduction. Repeated roentgenograms should be performed approximately every four to six months with the brace removed to follow the progression of the curve. The brace should be removed for a minimum of 12 to 24 hours before roentgenograms are taken so that the spine can go back to its deformed position and imaging can accurately detect curve deterioration.

This is not, however, a clinical practice accepted by all surgeons. Another option is to assess patients at follow-up with roentgenograms taken with the brace on to monitor the effectiveness of the orthosis in controlling the deformity. This will allow for brace adjustments, which are often necessary as patients grow. Roentgenograms out of the brace would be only required in the evidence of curve progression despite compliant bracing or at completion of brace treatment to assess the true size of the deformity and make definitive decisions in terms of the need for surgery.

A progression of 6° or more during brace treatment or need for surgical stabilization is considered failure of brace treatment.

## HOURS PER DAY

Studies conducted on the number of hours per day of brace-wearing show that the more hours per day the brace is worn, the better the result. The brace is usually prescribed for full-time wear with time out for bathing, swimming, physical education and sport. The child should be encouraged to be active in sporting activities while continuing to wear the brace if possible. Contact sports are not allowed with the brace to protect other participants. These activities generally represent an average of two to four hours a day to ensure brace-wearing of 21 to 23 hours daily.

Use of the brace part-time or only at night has been advocated by some physicians and is widely used in some institutions. However, there is a paucity of long-term follow-up data to prove the effectiveness of this wearing regimen in adolescents, and all series on effective orthotic treatment were with full-time wear. Some small series with a short follow-up after bracing suggest that part-time wear can be effective. However, these reports do not compare their results to natural history or full-time bracing.

Wiley *et al*. analyzed the results of bracing according to the wearing regimen.^26^ Patients were divided into noncompliant (less than 12 hours per day), part-time (between 12 and18 hours per day) brace-wearing and full-time brace-wearing (between 18 and 23 hours per day) groups. The initial curves were similar in the three groups. Patients who wore the brace less than 12 hours per day were associated with an average curve progression from 41.3° to 56.3°, and those who wore the brace part-time progressed from 37.6° to 41.2°. Significant curve improvement was noted in the full-time patient group, and curves measured 35.7° at final follow-up compared to 39.3° at brace-fitting. In addition, the surgical rate also depended on brace compliance, the rate being 73% in noncompliant patients compared to 9% in the fully compliant group.^26^

Green^27^ reported that 16 hours per day of bracing was effective in slowing curve progression. He studied a heterogeneous group of patients with curves between 23° and 49° and found that only 9% curves progressed 5° or more. However, both Boston and Milwaukee braces were used for treatment, and follow-up was limited. Similarly, Emans *et al*.^28^ found part-time brace-wear to be as effective as full-time wear for smaller curves. Allington and Bowen^29^ reported no difference in the efficacy of full-time versus part-time wear using the Wilmington brace for curves of 30° to 40°, but observed that 58% of patients progressed more than 5° degrees in the brace. Peltonen *et al*.^30^ also noted that the results of 12 hours per day of bracing were similar to the results of 23 hours per day of bracing.

## WHEN TO STOP TREATMENT

Brace-weaning begins when the patient reaches skeletal maturity, determined as the finding of a Risser sign of 4, i.e., more than 12 months post-menarche and lack of growth in height. Over a period of two to three months, the time of brace-wear is decreased progressively, and a roentgenogram is then performed of the patient without the brace. If the spine remains stable, brace-weaning continues over another two to three months with a further progressive decrease in brace-wear. After the second phase of weaning, another roentgenogram without the brace is performed to verify the stability of the spine. If stability is maintained, the weaning program continues until the patient is completely independent of the brace. On the other hand, if the brace is weaned off and there is deterioration of the residual curve, this may constitute an indication for surgical correction of the scoliosis. If the patient is skeletally mature, there is no evidence to support that continuing bracing regime provides any treatment benefit.

## COMPLICATIONS OF BRACE TREATMENT

Brace treatments have some disadvantages. Treatment with a brace can be rather bothersome.^31^,^32^ Patients, usually young adolescents aged between 10 and 16 years, have to wear the brace for 18 to 23 hours a day for several years, the brace is often visible and can be uncomfortable to wear.^33^,^34^ Moreover, noncompliance with brace-wear is often an issue and varies from refusal to wear the orthosis, to premature discontinuation of the use of that brace, to less than full-time use of the brace. Lack of compliance is related to several factors, including the unacceptable appearance of the brace to the body image conscious teenager, and the discomfort from chin and throat contact (especially Milwaukee brace) or from the pelvic or axillary portion of the brace, especially Thoraco-lumbo-sacral orthosisbraces (TLSO). A recent study showed that scoliosis patients are willing to undergo brace treatment only if it provides sizeable reduction of the risk of surgery.^34^ While some studies report little variation in compliance between Milwaukee brace and TLSO braces, other show significant less compliance with the Milwaukee brace when compared to TLSOs.^3^,^35^

Other problems encountered due to brace treatment include skin irritation, a temporary decrease in vital capacity, and mild chest wall and inferior rib deformation. Skin irritation is a common problem and more frequent in warm climates and during the summer months due to the increase in heat and sweat. To reduce the likelihood or occurrence of skin irritation, frequent changing of the cotton undergarment is recommended, but discontinuation of brace treatment due to skin irritation is uncommon. Vital capacity may be temporarily reduced in patients treated with thoraco-lumbo-sacral orthosis, and mild chest wall and inferior rib deformation can appear during treatment.

Chest wall and rib deformation commonly occurs if bracing is performed at ages where the chest is very plastic and easily deformed with drooping of the ribs on the convexity of the scoliosis, where corrective forces are applied. When brace use is discontinued, the mild deformity of rib cage usually disappears. However, if full-time bracing starts at very young age and continues for many years, chest wall and rib deformation may become permanent and may not reverse.^7^–^14^

## BRACE TYPES

### Cervico-thoraco-lumbo-sacral orthosis (Milwaukee brace)

The Milwaukee brace, also named cervico-thoraco-lumbo-sacral orthosis (CTLSO), is a full torso brace extending from the pelvis to the base of the skull.^15^–^19^ It was originally designed by Blount and Schmidt in 1946 for postoperative care when surgery required long periods of immobilization, and it has subsequently been used for thoracic and double curves. Milwaukee braces are often custom-made from a mould of the patient’s torso. This brace has one anterior and two posterior bars attached to a pelvic girdle made of leather or plastic, as well as a neck ring. The ring has an anterior throat mould and two posterior occipital pads, which fit behind the patient’s head. Lateral pads are strapped to the bars, and adjustment of these straps holds the spine in alignment.

Curve patterns that should be treated in a Milwaukee brace are thoracic curves that have an apex at or above T8, double thoracic, and other double curves when the apex of the thoracic component is above T8, i.e., double thoracic and lumbar, or double thoracic and thoracolumbar patterns.

*Success rate:* Curves between 20° and 29° with a Risser sign between 0 and 1 progressed 28% less than untreated curves of similar magnitude (40% versus 68%, respectively). Treated curves of similar magnitude but with a Risser sign of 2 or more progressed 13% less than untreated curves (10% versus 23%, respectively). Similarly, curves between 30° and 39° with a Risser sign between 0 and 1 progressed 14% less than untreated curves of similar magnitude (43% versus 57%, respectively). Treated curves of similar magnitude but with a Risser sign of 2 or more progressed 21% less than untreated curves (22% versus 43%, respectively).^3^,^15^

### Thoraco-lumbo-sacral orthosis

To improve patient compliance, substantially less bulky and lightweight thoraco-lumbo-sacral orthoses (TLSOs) were developed. TLSO is the generic name for a group of orthoses characterized by a pelvic portion similar to the pelvic section of the Milwaukee brace and an upper portion extending up to one or both axillae or only to the lower thoracic area. Although there are many variations in their design, generally named after the city or center of origin, they all function basically on the same principle. This type of brace is generally prescribed for lumbar and thoracolumbar curves, and thoracic curves with an apex at or below T8.

### Boston brace

Hall and Miller jointly created the Boston brace in 1972^33^ ; and Watson, Hall and Stanish first reported on its efficacy in 1977.^35^ The brace opens at the back and corrects curvatures by pushing the spine with small pads placed against the ribs, which are also used for partial rotational correction. These pads are usually placed in the back corners of the brace so that the body is thrust forward against the front of the brace, which acts to hold the body upright. Areas of relief are provided opposite the sites of corrective force to allow the patient to pull the spine away by active muscular effort.^28^ The brace also has a 15° lumbar lordosis built into it. The brace runs from just above the seat of a chair (when a person is seated) to around shoulder blade height and is not particularly useful in correcting very high curves.^5^,^25^,^26^,^28^,^35^ In the early 1990s, the original brace design was modified to add 15° lumbar lordosis into the pelvic module to better de-rotate the spine.^33^

*Success rate:* The brace has been shown to be particularly effective for curves ranging from 20° to 59° between T8 and L2. At the beginning of treatment, brace correction is about 50%, decreasing to 15% by the time of brace discontinuance. With Boston brace treatment, approximately half (49%) of the curves remain unchanged, 39% are stabilized with a final correction of 5° to 15°, 4% are stabilized with a correction superior to 15°, 4% lose between 5° to 15°, and 3% progress more than 15°. A study by Emans *et al*. reported that 11% of patients underwent surgery during the period of bracing.^28^

### Wilmington brace

G. Dean MacEwen (1970) developed the Wilmington brace, also known as the DuPont brace. It is a custom-made, plastic, underarm thoraco-lumbo-sacral orthosis. The brace is a total contact orthosis and is designed as a body jacket, which opens in the front for easy removal and is held closed by adjustable straps. Similar to the Boston brace, it is not useful in correcting very high curves.^29^

*Success rate*: Progression of the deformity by 5° or more is generally observed in 36% of patients treated with full-time bracing for a curve of less than 30° degrees compared to 41% of patients managed with part-time bracing. Failure rates are higher in patients with curves between 30° and 40° managed with both full-time (58%) and part-time bracing (59%).^27^

### Lyon brace

The Lyon brace was designed by Stagnara (1947) and is also known as the Stagnara brace. It is composed of a pelvic section with axillary, thoracic and lumbar plates connected in units by two vertical aluminum rods, one anterior and one posterior. The pelvic section is composed of two lateral valves, one for each hemipelvis. The valves are connected by metal pieces to the vertical aluminum rods. Forces are applied at the two neutral vertebrae, and a counterforce is applied at the apex of the curve. It is usually prescribed for progressive scoliosis with lumbar or low thoracolumbar curves between 30° to 50°.^36^,^37^

*Success rate*: The overall reported efficacy of the Lyon brace is 95%. However, it drops to 87% for thoracic curves and to 80% in patients with Risser sign 0.^37^

### Chêneau brace

Jacques Chêneau designed the original Chêneau brace in 1979.^38^–^40^ The brace is commonly used for the treatment of scoliosis and thoracic hypokyphosis in many European countries, Israel and Russia. However, it is not commonly prescribed in North America and the United Kingdom. The Chêneau brace utilizes large, sweeping pads to push the body against its curve and into blown out spaces, and is usually coupled with the Schroth physical therapy method. The Schroth theory holds that the deformity can be corrected through retraining of muscles and nerves to learn what a straight spine feels like, and by breathing deeply into areas crushed by the curvature to help gain flexibility and expand.^38^,^39^ The brace helps patients to perform their exercises throughout the day. It is asymmetrical and used for patients of all degrees of severity and maturity, and often worn 20 to 23 hours daily. The brace principally contracts to allow for lateral and longitudinal rotation and movement.^40^

### Rigo-Chêneau system (RCS brace)

Rigo *et al*. have further developed the original Chêneau brace by designing the Rigo-Chêneau system (RCS) brace. The main indication are curves up to 60° (First grade scoliosis: angle up to 40°; and Second grade scoliosis, between 40° and 60°; according to the Rigo classification.^41^

### Malaga brace

The Malaga brace is a custom-made TLSO, commonly prescribed in southern Spain but relatively unknown outside that country. It is a corrective spinal orthosis used in the treatment of coronal plane curves, but with no de rotation element incorporated in the brace.

The brace is of monovalve construction with a posterior opening that closes with metal fasteners. The patient wears the brace for approximately 23 hours per day, and it is indicated for progressive curves between 20° and 30°.^42^

### SPoRT brace (also known as “Sforzesco” brace)

The SPoRT (symmetric, patient-oriented, rigid, three-dimensional active) brace is symmetrical and built with a plastic frame reinforced with aluminum rods. It has two lateral elements that cover the back from the pelvis to the armpits, and the abdomen. These are linked to a posterior, centrally located aluminum rod, and the brace closes anteriorly with straps on the abdomen and another transverse bar at the level of the manubrium sternalis. The brace corrects hip misalignments through padding. Large, sweeping, thick pads push the spine to a corrected position. To prevent over-correction, however, the brace also has “stop” pads to hold the spine from moving too far in the other direction. This brace is used for all curve patterns and types, even for those curves considered as too late for brace treatment by other methods. It is typically worn 22 hours a day and often coupled with a physical therapy program.^43^,^44^

*Success rate*: In terms of Cobb’s angle, most curves have been shown to remain stable or to slightly improve. The SPoRT brace developing team found that it is possible to obtain scoliosis correction similar to cast in the corrective phase of adolescent idiopathic scoliosis treatment.^44^

### Nighttime braces

Despite the development of low-profile TLSO, such as the Boston brace, full compliance with a brace program that demands 18 to 23 hours of daily wear through skeletal maturity is difficult for adolescents. Nighttime bracing systems were developed to improve patient compliance by reducing the total time in the brace and eliminating the social anxiety created by daytime wear.

Nighttime braces are more effective in patients with isolated, flexible thoracolumbar and lumbar curves. They are also recommended to patients noncompliant with a full-time wear program, patients in whom other types of orthotic management had failed, and patients nearing skeletal maturity who may not require full-time wear.^17^,^33^,^45^–^48^

There have been previous studies comparing a nighttime orthosis to more traditional methods.^17^,^49^–^51^ Katz *et al*., retrospectively recommended the use of the Boston brace in curves between 36° and 45° because it prevented curve progression of 6° or more in 57% of patients, as compared with only 17% success in using the Charleston orthosis.^17^ The Boston orthosis also controlled curves of 25° to 35° more effectively than did the Charleston orthosis, preventing progression in 71% of patients versus 53% using Charleston orthosis.^17^ Howard *et al*. also found that the TLSO was superior at preventing curve progression when compared with the Charleston brace.^49^ Gepstein *et al*.,^50^ however, found no statistical difference in the surgery rate of 13.5% using the TLSO when compared with surgery rate of 11% using the Charleston brace.^50^ Similarly, Janicki *et al*. found the Providence nighttime orthosis more effective in avoiding surgery and preventing curve progression than a TLSO in a comparable population of patients with adolescent idiopathic scoliosis having initial curves of 25° to 40°.^51^

### Charleston brace

The Charleston bending brace was designed with the idea that compliance would increase if the brace was worn only at night. Hooper and Reed^6^,^46^,^47^,^52^ collaborated in 1978 on the early development of this new side-bending brace for nocturnal wear.^17^,^46^,^47^,^49^,^50^,^52^ The orthosis is asymmetrical and fights against the body’s curve by over-correcting the deformity. It grips the hips much like the Boston brace and rises to approximately the same height, but pushes the patient’s body to the side. It is used in single, thoracolumbar curves in patients 12 to 14 years of age (before structural maturity) who have flexible curves in the range of 25° to 35°.^46^,^47^,^52^

*Success rate*: Patients with a curve over 25° and a Risser sign between 0 and 2 showed a rate of surgery between 12% and 17%.^46^,^47^,^50^ In a 2002 study, it has been shown to be equally effective as the Boston brace.^50^

### Providence brace

The Providence brace was developed by D’Amato, Griggs and McCoy in the mid-1990s.^53^ The brace works by the application of controlled, direct, lateral and rotational forces on the trunk to move the spine toward the midline or beyond the midline. It does not bend the spine as with the Charleston bending brace. The goal is to use the centerline as a reference and bring the apices of the scoliotic curve to that line or beyond through the application of lateral forces. This involves the use of three-point pressure systems and void areas that are located opposite these pressure points. Compared with natural history and on the basis of the prospective data from the study by Nachemson *et al*.,^2^ the Providence brace is found to be effective in preventing curve progression of deformities less than 35° and low apex curves of over 35°. It is more successful in curves with apex at or below T9 compared to curves with apex cephalad to T8.^48^,^53^

*Success rate*: Recent studies showed that the Providence night brace generally achieves an average of about 90% for brace correction of the primary curve; and during followup, progression of the curve of more than 5° should be expected in about 25% of cases. The night brace may be recommended for the treatment of adolescent idiopathic scoliosis with curves less than 35° in lumbar and thoracolumbar cases.^48^,^53^

### Soft brace: The SpineCor brace

The SpineCor brace was developed by Coillard and Rivard in the mid-1990s. The brace has a pelvic unit made of plastic, from which strong elastic bands wrap around the body, pulling against curves, rotations and imbalances. It is most successful when patients have relatively small and simple curvatures and are structurally young and compliant. The SpineCor bracing method is an adjustable, flexible and noninvasive technique providing correction that continues as a child moves and grows. The brace is usually worn 20 to 22 hours a day, and the patient can remove it for no more than two to three hours a day.

*Success rate*: A 2003 study reported that after two years, the SpineCor brace was able to correct scoliotic curves by 5° in 55% of patients. The remaining 45% were stabilized (38%) or worsened by more than 5° (7%). Recent studies demonstrated a trend different from the findings of the SpineCor developing team and reported a lower success rate than rigid spinal orthosis.^54^–^56^ According to Wong *et al*.,^54^ in patients with curves between 20° and 30° before skeletal maturity, a rigid brace showed better results than the elastic one in the follow-up at 45 months: 31.8% in the SpineCor group had 5° or more of curve progression versus 4.7% in rigid brace.

### Other conservative treatments

Opinions differ in the international literature on the efficacy of conservative approaches to scoliosis treatment. Alternative forms of nonsurgical treatment such as chiropractic or osteopathic manipulation, acupuncture, exercise or other manual treatments, or changes in diet and nutrition pattern have not yet been proven to be effective in controlling spinal deformities.

Although a subject of debate, most experts agree that physiotherapy alone will not affect the progression of a structural scoliosis. However, there is agreement that a selective physical therapy program in conjunction with brace treatment is beneficial. The triad of outpatient physiotherapy, intensive inpatient rehabilitation, and bracing has proven effective as a conservative mode of scoliosis treatment in central Europe.^38^,^39^

Acupuncture involves penetration of the skin by thin, solid, metallic needles that are stimulated either manually or electrically, and it is commonly used for pain control throughout the world, although the putative mechanisms are still unclear. To date, only one study has been published, and the effects of acupuncture in the treatment of patients with scoliosis require further investigation.^57^

Electrotherapy was hailed as a promising therapy but failed to alter the natural history of idiopathic scoliosis. With electrotherapy, the lateral muscles on the convexity of the curve are stimulated electrically. It has been shown that no benefit was observed in approximately half of the patients treated by nighttime electrotherapy and that the difference in progression between bracing programs and electrical stimulation was significant.^29^,^58^

## CONCLUSION

Brace treatment is the only method that has been proven to alter the natural history of idiopathic scoliosis. However, different orthoses and many bracing regimens exist. Observation is appropriate for small curves, whereas bracing is generally indicated for progressive curves or for curves over 29° in a skeletally immature child. Braces are generally prescribed for more than 20 hours a day, and the results of brace treatment correlate to treatment compliance. Problems encountered with bracing are limited.
